# Effect of the cross-linker length of thiophene units on photocatalytic hydrogen production of triazine-based conjugated microporous polymers†

**DOI:** 10.1039/d1ra07916f

**Published:** 2021-12-23

**Authors:** Xiao Han, Yan Zhang, YunYun Dong, Jinsheng Zhao, Shouli Ming, Junhong Zhang

**Affiliations:** College of Chemistry and Chemical Engineering, Liaocheng University Liaocheng 252059 P. R. China j.s.zhao@163.com mingshouli@126.com zhangjunhong@lcu.edu.cn; Shandong Provincial Key Laboratory of Chemical Energy Storage and Novel Cell Technology, Liaocheng University Liaocheng 252059 PR China

## Abstract

Conjugated microporous polymers (CMPs) have been investigated in the field of photocatalytic hydrogen production because of their extended π-conjugation, tunable chemical structure and excellent thermal stability. Herein, we construct three CMPs based on thiophenes and triazine, and prove the effect of cross-linker length on photocatalytic activity of CMPs. BTPT-CMP1 exhibits blue-shifted optical absorption compared to BTPT-CMP2 and BTPT-CMP3 with long cross-linkers, however, possesses higher photocurrent because of the large specific surface area and small interface charge transfer resistance of BTPT-CMP1. It was found that BTPT-CMP1 (5561.87 μmol g^−1^ h^−1^) with short cross-linkers exhibits better photocatalytic performance compared to BTPT-CMP2 (1840.86 μmol g^−1^ h^−1^) and BTPT-CMP3 (1600.48 μmol g^−1^ h^−1^). Also, BTPT-CMP1 possesses a higher hydrogen evolution rate than most reported 1,3,5-triazine based conjugated polymers. These results demonstrate that the cross-linker length has great influence on the photocatalytic properties of conjugated microporous polymers, which offers theoretical direction for designing high-performance CMPs.

## Abstract 2

Conjugated microporous polymers (CMPs) have been investigated in the field of photocatalytic hydrogen production because of their extended π-conjugation, tunable chemical structure and excellent thermal stability.

## Graphical abstract

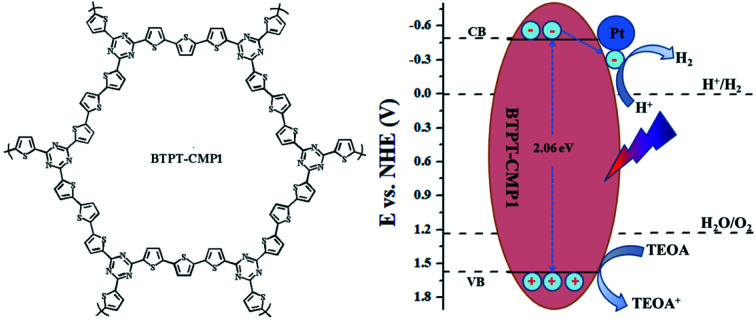

## 1. Introduction

Environmental pollution and the fossil energy crisis have become the two most concerning issues in the 21st century.^1,2^ Hydrogen energy, as a recognized clean energy with no pollution, has attracted increasing attention from society. Photocatalytic hydrogen evolution was firstly proposed by Honda and Fujishima in 1972 based on the potential applications of hydrogen, which has become a research hotspot in the academic field.^3,4^ In the last few decades, research mainly focused on metal-based inorganic semiconductor photocatalysts including TiO_2_, ZnO, CdS, GaP, and SiC.^5,6^ However, metal-based inorganic semiconductor photocatalysts face many challenges, such as low photocatalytic activity, poor charge separation ability, high toxicity and the limitation of rare metals reserve.^7,8^ Compared to inorganic semiconductors, organic polymers have many advantages, such as easily-controllable molecular structure and performance, rich raw material reserves, light weight, *etc.*^9–11^ The research on organic polymers used as photocatalysts began in the 1980s. After that, various types of organic polymers for photocatalytic hydrogen evolution have been widely studied, such as conjugated microporous polymers (CMPs), linear conjugated polymers and covalent organic framework materials, *etc.*^12–14^ Among them, CMPs is emerging class of organic porous materials with large π-conjugated framework and many nanoscale pores.^15–17^ More importantly, the energy band structure, optical absorption and pore size of CMPs can be fine-tuned by changing the chemical structure. These distinguished traits make CMPs have large potential applications in the field of photocatalysis.^17–20^

In order to obtain high-performance CMPs used in photocatalytic hydrogen production, various strategies have been proposed, such as extending conjugated block, modifying side chain structure, constructing donor–acceptor (D–A) type molecular structure, *etc.*^21,22^ For example, Wang *et al.*^23^ reported the dibenzothiophene dioxide-containing CMPs with cross-linkers from benzene to biphenyl, and to *p*-terphenyl, and investigated the effect of cross-linker length on photocatalytic property. Lin *et al.*^24^ reported a truxene-based conjugated polymer with hydrophilic amino side chains, which dramatically modified the wettability and hydrogen evolution rate of polymer than that with hydrophobic octyl chains. Shu *et al.*^25^ studied the influence of molecular geometry on photocatalytic property. They constructed two bisulphone-containing conjugated polymers with 1D and 3D molecular structures, which suggest that the linear polymers exhibit increased photocatalytic performance related to that with 3D molecular structure due to fast charge transport in 1D molecular skeleton. Lan *et al.*^26^ prepared a series of D–A type conjugated polymers based on dibenzothiophene-*S*,*S*-dioxide (FSO) as the acceptor unit and pyrene as the donor unit. They found that the introduction of FSO can widen the optical absorption region of polymer and improve the exciton-separation efficiency. Zhao *et al.*^27^ also obtained two kinds of FSO based CMPs photocatalysts and investigated the effect of linking sites on the photocatalytic hydrogen evolution. To further widen the optical absorption, Xiao *et al.*^28^ used diketopyrrolopyrrole as acceptor unit to construct D–A type CMPs photocatalyst. These results suggest that D–A type conjugated polymers not only regulate the optical absorption, but also are helpful for exciton separation under visible light. The conjugated D–A type CMPs could enable efficient exciton separation, however, the dissociated electron and hole often recombine simultaneously. In order to suppress electron and hole recombination, Guo *et al.* constructed a series of D–A_1_–A_2_ type CMPs comprising two kind of acceptor units with different energy levels.^29^ In short, various strategies have been proposed to construct CMPs with high hydrogen evolution efficiency, however, the photocatalytic parameters of CMPs can't meet the standards of commercial application. Therefore, identifying the key factors affecting the hydrogen evolution efficiency of CMPs is of great significance for the design of high-performance materials.^30,31^

**Fig. 1:**
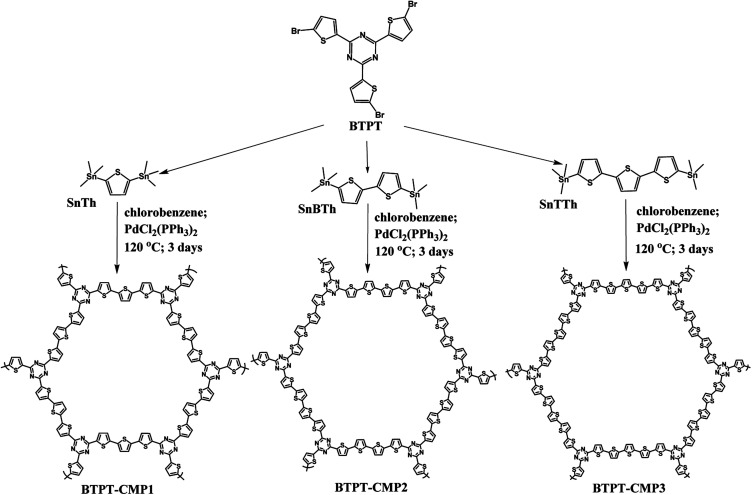
Synthesis routes of BTPT-CMP1, BTPT-CMP2 and BTPT-CMP3.

Recent research indicate that triazine is an appropriate acceptor unit for the construction of D–A type CMPs applied in photocatalytic hydrogen evolution due to the hydrophilic nature of nitrogen atoms in triazine unit.^32,33^ In this study, we synthesized three thiophene-triazine based D–A type CMPs by Stille coupling reaction, as shown in Fig. 1. The difference of three CMPs is the cross-linker from monothiophene to bithiophene and *p*-terthiophene. As we known that polythiophene and its derivatives could be n-doped by ions under negative potential window, which might be helpful to charge transport.^34^ It is speculated that thiophene-triazine based D–A type CMPs possess high charge transport rate, further produce more photocurrent for photocatalytic hydrogen evolution.

Herein, the optical and electrochemical properties of the three CMPs were investigated comparatively. BTPT-CMP1 possesses much higher photocurrent relative to BTPT-CMP2 and BTPT-CMP3 due to fast transport rate and less recombination of photogenerated charges in BTPT-CMP1. The results indicate that the hydrogen production performance of the prepared CMPs decrease gradually with increase of the cross-linker length. BTPT-CMP1 with monothiophene cross-linker displays the hydrogen-producing rate of 5561.87 μmol h^−1^ g^−1^ under visible light with 3 wt% Pt loaded. Also, BTPT-CMP1 keeps good stability over 15 h without obvious photocatalytic efficiency loss. These results demonstrate that BTPT-CMP1 possess large application potential in photocatalytic hydrogen evolution.

## 2. Experimental section

### 2.1. Materials and the characterization methods

The chemical agents except 2,4,6-tris(5-bromothiophene-2-yl)-1,3,5-triazine (BTPT) are obtained from the commercial channels, and BTPT was synthesized according to our previous report.^35^ All of the detailed information is given in the ESI.† And also, characterization methods for the polymer materials and the related apparatus are given in the ESI.† The detailed information on the hydrogen evolution activity of the polymers are also given in the ESI.†

### 2.2. Synthesis of BTPT-CMP1

BTPT-CMP1 was prepared by the Stille coupling reaction under nitrogen atmosphere (Fig. 1). Typically, 200 mg of BTPT (354.52 μmol) and 217.90 mg of SnTh (531.79 μmol) were dispersed into chlorobenzene under stirring condition. Then, 18.7 mg of PdCl_2_(PPh_3_)_2_ (26.9 μmol) was added to the Schlenk tube. The mixed solution was heated under nitrogen at 120 °C for 72 h. After reaction, the red powder precipitate was obtained, followed by washed with ethanol and acetone, finally vacuum dried at 70 °C for 12 h. The product mass: 132.50 mg, yield: 83%.

### 2.3. Synthesis of BTPT-CMP2

200.00 mg of BTPT (354.52 μmol) and SnBTh (261.57 mg, 531.79 μmol) were firstly dispersed into chlorobenzene (35 mL). Then 18.7 mg of PdCl_2_(PPh_3_)_2_ (26.9 μmol) was added as the catalyst to the Schlenk tube under a N_2_ atmosphere. Then, the reaction solution was stirred for 3 days at 120 °C. After reaction, the red powder precipitate was obtained, followed by washed with ethanol and acetone, finally vacuum dried at 70 °C for 12 h. The product mass: 164.70 mg, yield: 81%.

### 2.4. Synthesis of BTPT-CMP3

BTPT-CMP3 was synthesized according to the preparation method of BTPT-CMP2, except that SnTTh was used as the organotin compound for the Stille reaction. The product mass: 197.60 mg, yield: 80%.

## 3. Results and discussion

### 3.1. Synthesis and characterization of CMPs

**Fig. 2:**
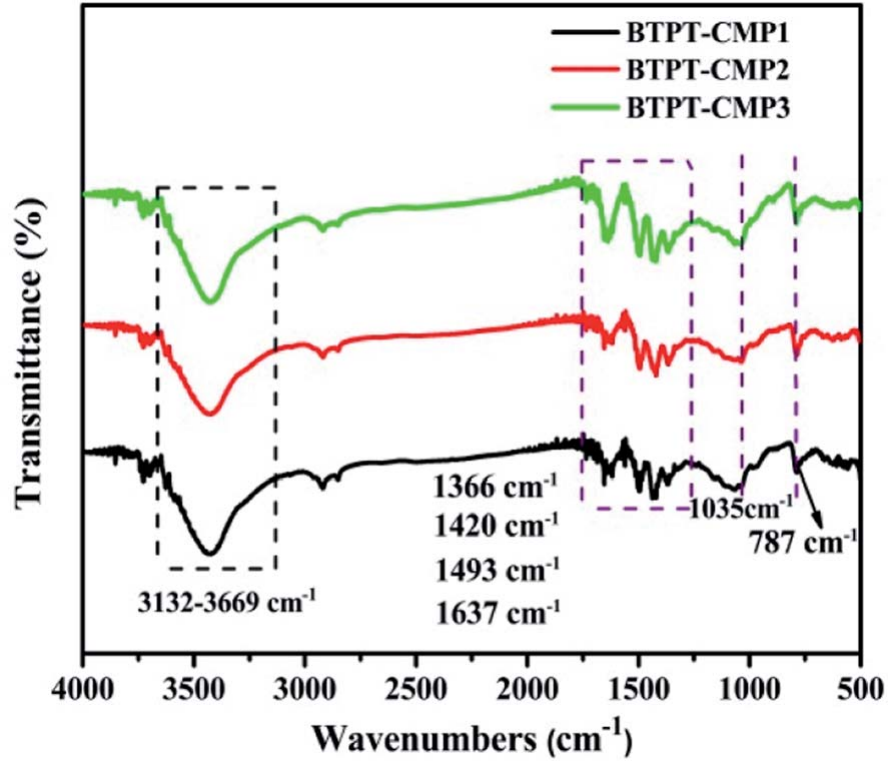
FT-IR spectra of the polymers.

**Fig. 3:**
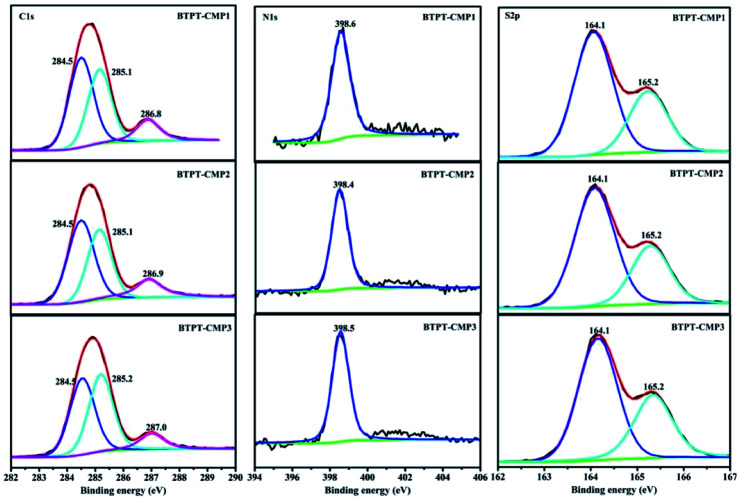
High-resolution XPS spectra of the polymers.

The triazine compound (BTPT) was synthesized according to previous literature.^35^ The three thiophene based CMPs photocatalysts were prepared at satisfactory yields by the Stille coupling reaction. These polymers exhibit poor solubility in common organic solvents because of their rigid backbones and the cross-linked networks.^19^ The structures of the polymers were proved by FT-IR and solid-state ^13^C CP-MAS NMR. As shown in Fig. 2, a strong peak at 1493 cm^−1^ is found, which can be ascribed to the aromatic C–N stretching vibration of the triazine ring.^36,37^ The characteristic peak located at 1420 cm^−1^ and 1637 cm^−1^ originate from the skeleton vibrations of aromatic rings and thiophene rings. Besides, the characteristic vibration of the C–H in-plane bending (1035 cm^−1^) and C–H out-of-plane bending (787 cm^−1^) on the thiophene groups could be clearly observed. In addition, the peak at 1366 cm^−1^ is attributed to the O–H of physisorbed water, which is also proved by the broad band in the range of 3132–3669 cm^−1^. The solid-state ^13^C CP-MAS NMR was employed to further confirm their structures (Fig. S1†). The chemical shift at about 166 ppm is assigned to C atom on the triazine ring. The signals at 123–143 ppm are ascribed to C atoms on the thiophene units. Further structural details about polymers were investigated by X-ray photoelectron spectroscopy (XPS), as shown in Fig. 3. XPS spectra indicate there are carbon, nitrogen and sulfur in the three polymers, which is also proved by element mapping (Fig. S2 and S3†). For BTPT-CMP1, the C 1s spectrum exhibit two obvious peaks at 284.5 and 286.8 eV, which are assigned to the C=C and C–S in the thiophene rings, respectively. The peak located at 285.1 eV is ascribed to the C–N in triazine groups, which is also proved by the peak (398.6 eV) in N 1s spectrum. The S 2p spectrum is divided into two main peaks at 164.1 eV and 165.2 eV, corresponding to the S 2p_3/2_ and S 2p_1/2_ binding energies, respectively. As BTPT-CMP1, BTPT-CMP2 and BTPT-CMP3 exhibit similar XPS spectra.

**Fig. 4:**
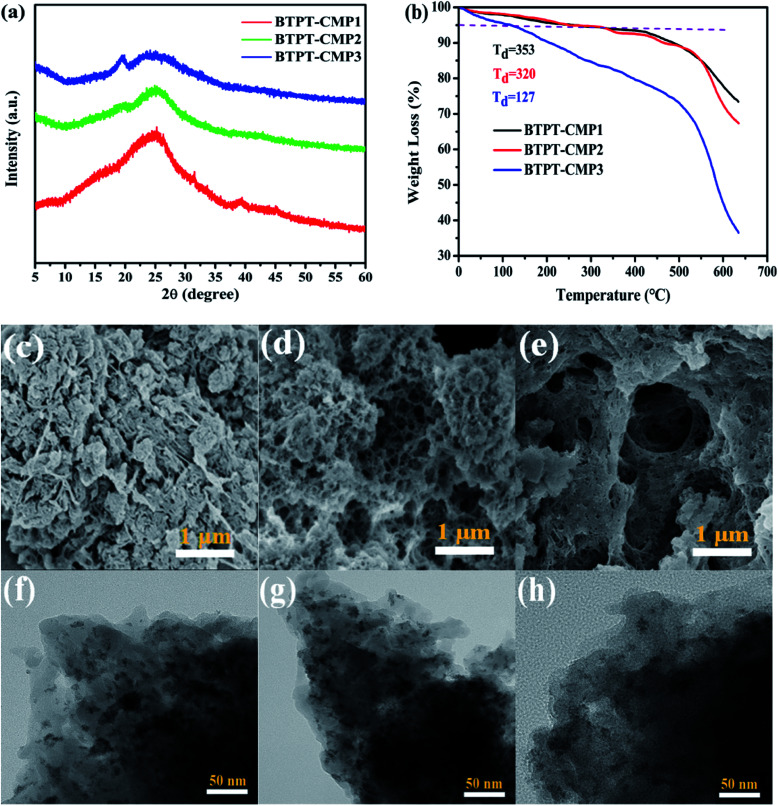
(a) PXRD patterns of the polymers; (b) TGA curves of polymers; SEM images of BTPT-CMP1 (c), BTPT-CMP2 (d) and BTPT-CMP3 (e); TEM images of BTPT-CMP1 (f), BTPT-CMP2 (g) and BTPT-CMP3 (h).

The crystalline structure and aggregation state of polymer could be illustrated with powder X-ray diffraction (PXRD). The broad diffraction peaks were observed in the PXRD as shown in Fig. 4a, which revealed the amorphous structure of the polymers. Also, the thermal stability of the three polymers were measured in nitrogen atmosphere. Thermal gravimetric analysis (TGA) indicate that the initial decomposition temperature (*T*_d_) for BTPT-CMP1, BTPT-CMP2 and BTPT-CMP3 are 353 °C, 320 °C and 127 °C, respectively (Fig. 4b). It is observed that BTPT-CMP1 and BTPT-CMP2 exhibit better thermal stability than BTPT-CMP3.

**Fig. 5:**
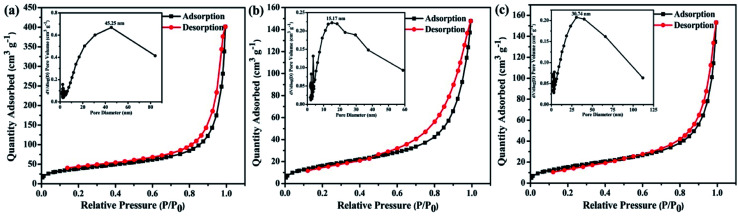
N_2_ adsorption–desorption isotherms of (a) BTPT-CMP1; (b) BTPT-CMP2; (c) BTPT-CMP3 at 77 K. Pore size distribution are shown in the insets.

The morphology of the polymers were investigated by scanning electron microscope (SEM) and transmission electron microscopy (TEM). The three polymers have sponge-like structure with obvious macropores and mesopores, as shown in Fig. 4c–e and f–h. The porous structure make polymer possess more specific surface area, which could provide more catalytic sites. The porosity of polymers were investigated by N_2_ adsorption–desorption experiment at 77 K (Fig. 5). The three polymers display faint N_2_-adsorption behavior at low relative pressure. The rapid nitrogen adsorption at very high relative pressure could be observed, which suggest that the three polymers possess obvious meso- and macroporous structures.^38^ The result is consistent with the findings of SEM. Also, the Brunauer–Emmett–Teller (BET) surface area of BTPT-CMP1, BTPT-CMP2 and BTPT-CMP3 were evaluated to be 138.4, 62.1 and 58.8 m^2^ g^−1^, respectively. Significantly, the specific surface area of BTPT-CMP1 is beyond 2 times higher than that of BTPT-CMP2 and BTPT-CMP3. The specific surface area difference might be related to polymeric cross-linker length. The phenomenon is usually observed in reported polymers containing different length cross-linkers.^39,40^ Also, the pore-size analysis of the three polymers was carried out, as shown in the inset of Fig. 5. All the three polymers possess wide pore-size distribution and the pore diameter concentrate into 45.25 nm for BTPT-CMP1, 15.17 nm for BTPT-CMP2 and 30.74 nm for BTPT-CMP3. The wide pore-size distribution of BTPT-CMP1 could be explained that derived from the interparticle porosities or voids.^41^ The large specific surface area and wide pore-size distribution are beneficial to provide more active sites for BTPT-CMP1, which is conducive to the increase of photocatalytic hydrogen production rate.

### 3.2. The optical and electric properties of CMPs

**Fig. 6:**
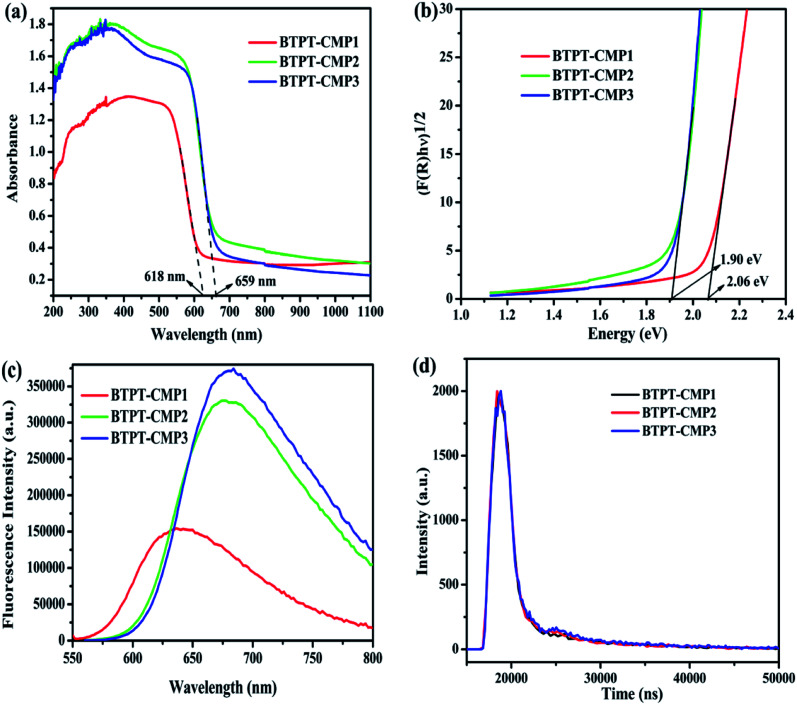
UV-Vis diffuse reflectance spectra (a) and band gaps (b) of polymers; photoluminescence spectra (c) and time-resolved photoluminescence spectra (d) of polymers at the excitation wavelength of 544 nm.

The UV-Vis diffuse reflectance spectra (DRS) of the three polymers are displayed in Fig. 6a, which reveal broad absorption from 200 nm to 650 nm. It is observed that BTPT-CMP2 and BTPT-CMP3 exhibit red-shifted absorption bands compared to BTPT-CMP1, which result from BTPT-CMP2 and BTPT-CMP3 possess larger conjugated molecular skeleton than BTPT-CMP1.^22^ In theory, BTPT-CMP3 possess larger conjugated molecular skeleton relative to BTPT-CMP2, however, no obvious optical absorption shift could be observed. The phenomenon might be ascribed to the poor solubility of BTPT-CMP3 in polymerization system restrict the formation of polymer with large conjugated molecular skeleton, which lead to BTPT-CMP2 and BTPT-CMP3 possess similar conjugation degree. Based on the absorption spectra of the three polymers, the band gaps are calculated to be 2.06 eV for BTPT-CMP1, 1.90 eV for BTPT-CMP2 and BTPT-CMP3 (Fig. 6b). In short, all the three polymers reveal broad absorption and narrow ban gap due to the electron push–pull effect of the D–A structure for polymer.^42^

As is well known, photoluminescence (PL) spectrum is one of the effective methods to evaluate the separation/recombination efficiency of photogenerated carriers. Therefore, the photoluminescence spectra of the three polymers were measured comparatively (Fig. 6c). As expected, BTPT-CMP1 (640 nm) exhibits blue-shifted emission peak relative to BTPT-CMP2 (680 nm) and BTPT-CMP3 (684 nm). However, it is observed that BTPT-CMP1 possesses much weaker emission intensity compared with BTPT-CMP2 and BTPT-CMP3, indicating less recombination of photogenerated electron and hole in BTPT-CMP1.^43,44^ Also, the time-resolved fluorescence decay spectra of the three polymers were studied to measure photogenerated electron lifetime. As shown in Fig. 6d, according to the fitting results, the emission lifetimes of BTPT-CMP1 (*τ*_1_ = 0.93 μs, *τ*_2_ = 8.68 μs) are much shorter than those of BTPT-CMP2 (*τ*_1_ = 0.97 μs, *τ*_2_ = 8.88 μs) and BTPT-CMP3 (*τ*_1_ = 0.98 μs, *τ*_2_ = 9.01 μs). Based on the decay fitting data, the average PL lifetime (*τ*_avg_) was calculated using the equation:
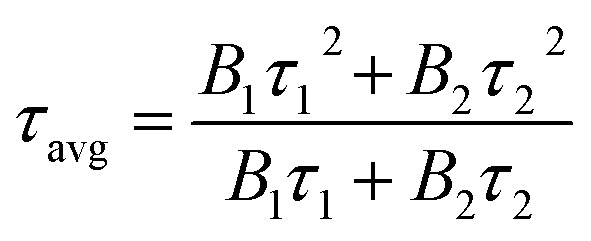


As a result, BTPT-CMP1 shows a *τ*_avg_ (7.63 μs), which is lower than that of BTPT-CMP2 (7.87 μs) and BTPT-CMP3 (8.16 μs), suggesting the higher activity of photogenerated electron in BTPT-CMP1. According to these results, it could be inferred that BTPT-CMP1 might possess better photocatalytic activity than BTPT-CMP2 and BTPT-CMP3.

**Fig. 7:**
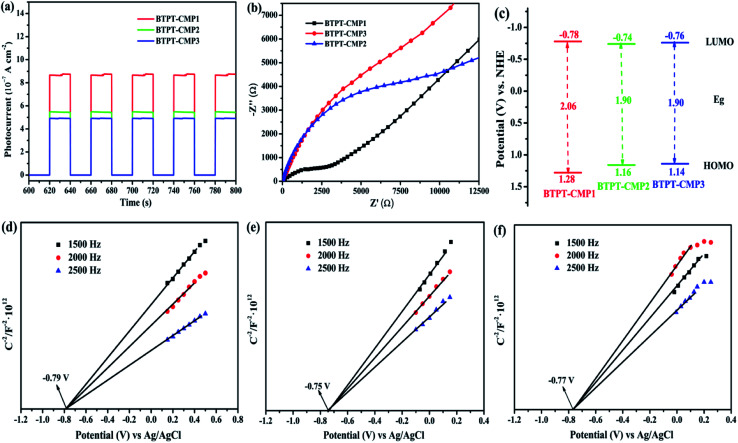
(a) Transient photocurrent and (b) Nyquist impedance plots of polymers measured in 0.5 M Na_2_SO_4_; (c) the band structure diagram of polymers; Mott–Schottky plots of BTPT-CMP1 (d), BTPT-CMP2 (e) and BTPT-CMP3 (f) at different frequency in an aqueous solution of Na_2_SO_4_ (0.5 M).

The separation efficiency and transport properties of photo-generated carriers in polymers were also characterized by photoelectrochemical measurement. As shown in Fig. 7a, the transient photocurrents under light irradiation are 0.87 μA cm^−2^ for BTPT-CMP1, 0.54 μA cm^−2^ for BTPT-CMP2 and 0.49 μA cm^−2^ for BTPT-CMP3, respectively. BTPT-CMP1 possesses much higher photocurrent than BTPT-CMP2 and BTPT-CMP3, implying that BTPT-CMP1 could produce more photo-generated electrons for photocatalytic reaction,^45,46^ which is consistent with the result of PL spectra. The larger specific surface area may be an critical reason to enhance the photocurrent of BTPT-CMP1 (Fig. 5).^47,48^

The three prepared polymers possess similar molecular structures, however, PL spectra and photoelectrochemical measurement indicate that BTPT-CMP1 could produce more photo-generated electrons relative to BTPT-CMP2 and BTPT-CMP3. To investigate the reasons for the differences, electrochemical impedance spectroscopy (EIS) measurement of the three polymers was performed from 100 000 Hz to 0.01 Hz. BTPT-CMP1 depicts smaller semicircle arc compared with BTPT-CMP2 and BTPT-CMP3 (Fig. 7b), which suggests little interface charge transfer resistance of BTPT-CMP1. As we known that little interface charge transfer resistance of polymer is beneficial to photo-generated electrons transport, further to activate reaction substrates.^49^

One important factor to determine the photocatalytic ability is the energy levels of conjugated polymers. Herein, the energy levels of the three polymers were characterized with the help of the cyclic voltammetry curves (Fig. S4†) and the analysis on Mott–Schottky plots. The HOMO and LUMO energy levels of the polymers can be calculated by employing the following equations:

1 LUMO = −e(*E*_onset,re_ + 4.8 − 0.50)

2 HOMO = LUMO − *E*_g_

In the equation, the value 0.50 (V) is the *E*_1/2_ of the ferrocene/ferrocenium (Fc/Fc^+^) *versus* Ag/AgCl electrode, and the 4.8 (eV) was the energy level of ferrocene/ferrocenium (*E*_Fc/Fc^+^_) *versus* vacuum, the parameter *E*_g_ refers to the optical bandgaps obtained from the Tauc plots as shown in Fig. 6b.^46^ The results reveal that LUMO levels are −0.78 V (−3.66 eV *versus* vacuum) for BTPT-CMP1, −0.74 V (−3.7 eV *versus* vacuum) for BTPT-CMP2 and −0.76 V (−3.68 eV *versus* vacuum) for BTPT-CMP3 *vs.* NHE, and HOMO are 1.28 V (−5.72 eV *versus* vacuum) for BTPT-CMP1, 1.16 V (−5.6 eV) for BTPT-CMP2 and 1.14 V (−5.58 eV) for BTPT-CMP3 *vs.* NHE (Fig. 7c and Table 1). All the three polymers possess enough driving forces for water-splitting. As shown in Fig. 7d, BTPT-CMP1 exhibits higher LUMO than BTPT-CMP2 and BTPT-CMP3, implying the larger driving force of BTPT-CMP1 to provide free electron activating H^+^. Also, Mott–Schottky plots analysis indicate that the flat band position (*V*_fb_) of BTPT-CMP1, BTPT-CMP2 and BTPT-CMP3 are approximately −0.79 V, −0.75 V and −0.77 V *vs.* Ag/AgCl (Fig. 7d–f), corresponding to −0.58 V, −0.54 V and −0.56 V *vs.* NHE, which equals to the Fermi level (*E*_F_) for the n-type semiconductor. The conduction bands of BTPT-CMP1, BTPT-CMP2 and BTPT-CMP3 can be calculated as −0.78, −0.74 and −0.76 V, respectively.^33^ It is also proved that BTPT-CMP1 possesses more negative LUMO than BTPT-CMP2 and BTPT-CMP3, which is consistent with the above electrochemical result.

**Table 1:** Photophysical properties and hydrogen evolution rate of polymers

Polymer	*S*_BET_ a (m^2^ g^−1^)	*λ*_onset_ b (nm)	HOMO^c^ (V)	LUMO^c^ (V)	*E*_g_ d (eV)	HER^e^ (μmol g^−1^ h^−1^)
BTPT-CMP1	138.4	618	1.28	−0.78	2.06	5561.87
BTPT-CMP2	62.1	659	1.16	−0.74	1.90	1840.86
BTPT-CMP3	58.8	659	1.14	−0.76	1.90	1600.48

a Specific surface area calculated from the N_2_ sorption-desorption isotherm.

b Initial optical absorption.

c Energy level *vs.* NHE calculated from CV curve.

d The optical bandgap obtained by the plot of (*αhν*)^2^ against *hν* in UV-Vis reflectance spectroscopy.

e 50 mg sample with 3 wt% Pt under visible light (*λ* > 420 nm).

### 3.3. The photocatalytic performance of CMPs

**Fig. 8:**
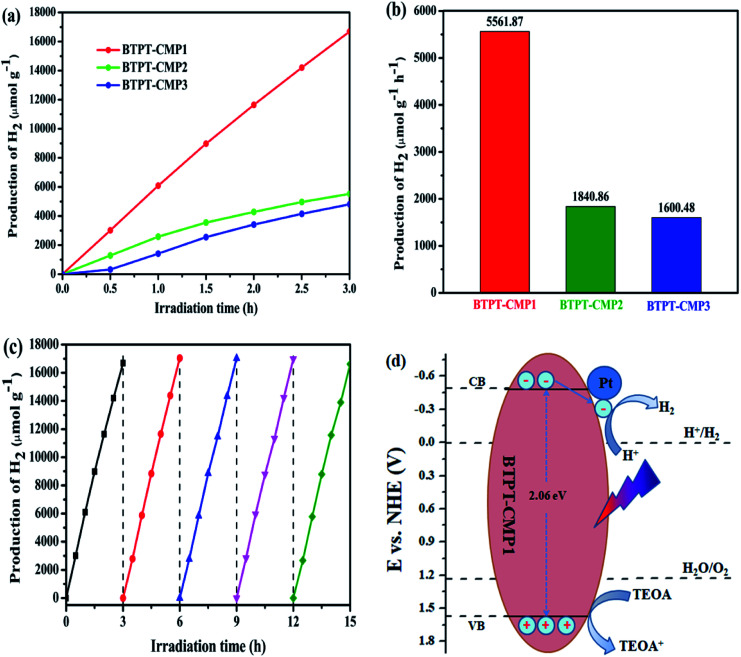
(a) Hydrogen evolution of the three polymers with 3 wt% Pt in a 5 : 1 mixture of H_2_O/TEOA under visible light (*λ* > 420 nm); (b) the H_2_ production rates of the three polymers; (c) cycling stability of BTPT-CMP1; (d) photocatalytic mechanism of BTPT-CMP1.

The photocatalytic H_2_-production activities was evaluated by using 50 mg samples with 3 wt% Pt in a 5 : 1 (v/v) mixture of H_2_O/TEOA under visible light. Fig. 8a records the H_2_ production activity of BTPT-CMP1, BTPT-CMP2 and BTPT-CMP3 within 3 h under the same conditions. The amount of photocatalytic H_2_ evolution are 16 685.6 μmol g^−1^ for BTPT-CMP1, 5522.5 μmol g^−1^ for BTPT-CMP2 and 4801.4 μmol g^−1^ BTPT-CMP3, respectively. The H_2_ production rate of BTPT-CMP1 is 3.02 times higher than that of BTPT-CMP2 and 3.47 times higher than that of BTPT-CMP3. The corresponding H_2_ evolution rates of the samples are presented in Fig. 8b. The corresponding H_2_ evolution rates of BTPT-CMP1, BTPT-CMP2 and BTPT-CMP3 are 5561.87 μmol g^−1^ h^−1^, 1840.86 μmol g^−1^ h^−1^ and 1600.48 μmol g^−1^ h^−1^, respectively. It could be seen that the H_2_ evolution rates of polymers decreased gradually with the increase of molecular cross-linker length. The better H_2_ evolution efficiency of BTPT-CMP1 could be ascribed to the following reasons: (1) the larger specific surface area and wider pore-size distribution are beneficial to provide more active sites for BTPT-CMP1; (2) little interface charge transfer resistance of BTPT-CMP1 help photo-generated electrons transport, and further activate more reaction substrates.

Significantly, 5561.87 μmol g^−1^ h^−1^ H_2_ production rate of BTPT-CMP1 is one of the most optimum values among the conjugated polymers. To assess the photocatalytic hydrogen production efficiency of BTPT-CMP1 comprehensively, triazine based conjugated polymers have been list in Table 2. BTPT-CMP1 exhibits higher hydrogen evolution rate than the most 1,3,5-triazine based conjugated polymers, which suggest the great application prospect of BTPT-CMP1 in photocatalysis field.

**Table 2:** Photocatalytic performance of polymers based on triazine

Photocatalyst	Cocatalyst content	HER (μmol g^−1^ h^−1^)	Reference
BTPT-CMP1	3 wt% Pt	5561	This work
Cl-ECF	3 wt% Pt	1296	50
CTF-2	3 wt% Pt	296	51
THT-TA	3 wt% Pt	246	52
P1	3 wt% Pt	1000	53
PCTF-1	1 wt% Pt	475	54
ZrTTA-6SH-ZnTFPP	3 wt% Pt	175	55
CTFS_10_	3 wt% Pt	2000	56
CTF-1_10 min	3 wt% Pt	1072	57
CTF-N	2.11 wt% Pt	10 760	58
TFA-COF	3 wt% Pt	80	59

Considering the potential application prospect of BTPT-CMP1, its long-term photocatalytic stability experiment is carried out under same condition. As presented in Fig. 8c, a linearly increasing amount of H_2_ evolved during 15 h is obtained. No obvious deactivation is observed after five cycles, demonstrating that BTPT-CMP1 as photocatalyst keeps good stability during hydrogen production procedure. The apparent quantum yield (AQY) of polymers were further investigated by using various band-pass filter. As shown in Fig. S5,† the AQY of BTPT-CMP1 recorded at 405, 420, 450, 500, 550 and 630 nm were 3.6%, 3.8%, 3.5%, 3.1%, 2.5% and 0.9%, respectively. The maximum AQY of 3.8% was obtained at 420 nm, which matches well with the UV/Vis absorption spectrum of BTPT-CMP1. The phenomenon implies that optical absorption ability of BTPT-CMP1 has a big influence on the photocatalytic reaction for hydrogen generation. For BTPT-CMP2 and BTPT-CMP3, the AQY values show downward trend as the incident light wavelength increasing. In addition, the AQY values of BTPT-CMP2 and BTPT-CMP3 are lower than that of BTPT-CMP1, which is in agreement with photocatalytic performance change. Also, XRD, FT-IR, UV-Vis spectra, SEM and TEM of BTPT-CMP1 before and after irradiation were characterized, as shown in Fig. S6–S9.† By comparing, there is no obvious structure difference for BTPT-CMP1 before and after irradiation, demonstrating its good structure stability.

### 3.4. The photocatalytic mechanism of CMPs

The photocatalytic reaction mechanism of BTPT-CMP1 is proposed according to these results, as displayed in Fig. 8d. During the photocatalytic reaction process, the conjugated microporous polymer absorb photons to generate excitons (electron and hole pairs). Then, the photo-induced electrons and holes transfer to CB and VB of BTPT-CMP1, respectively. Owing to the presence of heterojunctions between BTPT-CMP1 and Pt co-catalyst with lower Fermi energy level^25,60^ electron and hole pairs are directly split into free charges. The free electrons could transfer to Pt co-catalysts, and then react with H^+^ to evolve H_2_ directly. At the same time, the free holes on VB of the polymer react with TEOA to form oxidization product, TEOA^+^.

## 4. Conclusion

In conclusion, we have constructed three D–A type conjugated microporous polymers (CMPs) by using 1,3,5-triazine as the acceptor unit and thiophenes as the donor units, respectively. Due to the small conjugated skeleton, BTPT-CMP1 exhibits blue-shifted optical absorption than BTPT-CMP2 and BTPT-CMP3 with long cross-linkers. However, BTPT-CMP1 possess higher photocurrent than BTPT-CMP2 and BTPT-CMP3 due to the large specific surface area and little interface charge transfer resistance of BTPT-CMP1. An impressive hydrogen production rate of 5561.87 μmol g^−1^ h^−1^ was obtained by BTPT-CMP1, which is 3.02 times higher than that of BTPT-CMP2 and 3.47 times higher than that of BTPT-CMP3. Among the reported 1,3,5-triazine based conjugated polymers, BTPT-CMP1 exhibits higher hydrogen evolution rate than the most 1,3,5-triazine based conjugated polymers, which suggest the great application prospect of BTPT-CMP1 in photocatalysis field.

## Data availability

All related data can be available upon request.

## Conflicts of interest

There are no conflicts to declare.

## Supplementary Material

RA-012-D1RA07916F-s001
