# Supplementary material for: Effect of the cross-linker length of thiophene units on photocatalytic hydrogen production of triazine-based conjugated microporous polymers
Source: RSC Adv. 2021 Dec 23;12(2):708–18. doi: 10.1039/d1ra07916f (PMC8978623; doi:10.1039/d1ra07916f)
Supplement: RA-012-D1RA07916F-s001 [file RA-012-D1RA07916F-s001.pdf]

Electronic Supplementary Material (ESI) for RSC Advances.
This journal is © The Royal Society of Chemistry 2021

# Supporting Information

# Effect of Cross-linker Length of thiophene units on Photocatalytic Hydrogen Production of Triazine-Based Conjugated Microporous Polymers

Xiao Han[a], Yan Zhang[a], YunYun Dong[a], Jinsheng Zhao[a,b,*], Shouli Ming[a,*], Junhong Zhang[a,*]

[a]College of Chemistry and Chemical Engineering, Liaocheng University, Liaocheng, 252059, P.R. China.

[b]Shandong Provincial Key Laboratory of Chemical Energy Storage and Novel Cell Technology, Liaocheng University, Liaocheng, 252059, PR China

*Corresponding Author: j.s.zhao@163.com (J.S. Zhao); mingshouli@126.com (S.L. Ming); zhangjunhong@lcu.edu.cn (J.H. Zhang).

## Materials

2,5-bis(trimethylstannyl)thiophene (2SnTh); 5,5'-bis(trimethylstannyl)-2,2'-bithiophene (2SnBTh); 5,5''-bis(trimethylstannyl)-2,2':5',2''-terthiophene (2SnTTh) are purchased from SunaTech Inc. Bis(dibenzylideneacetone)palladium, tris(2-methylphenyl)phosphine and bis(triphenylphosphine)palladium(ii) chloride ($PdCl_2(PPh_3)_2$) are obtained from Alfachem (Zhengzhou, China). The 1-methyl-2-pyrrolidinone (NMP), triethanolamine (TEOA), ethanol, chloroform and chlorobenzene are bought from Aladdin Co., LTD. All reactants react without further purification. 2,4,6-Tris(5-bromothiophene-2-yl)-1,3,5-triazine (BTPT) was prepared according to the previous report [1].

## Characterization

Fourier transform infrared (FT-IR) spectra were measured on a Nicolet Avatar 360 FT-IR

spectrometer with KBr pellets. Solid state magic angle spinning $^{13}C$ CP-MAS NMR measurement was carried out on a Bruker Avance 400 model 600 MHz NMR spectrometer at a MAS rate of 10 kHz. X-ray photoelectron spectroscopy (XPS) was performed with ESCALAB 250Xi spectrometer. X-ray diffraction (XRD) was carried out with the 2θ range from 5 to 800 using Kigaka D/max 2500 X-ray advance diffractometer with a Cu-Ka radiation, and a step scan mode was adopted with a scanning step of 0.02. Morphology of CMPs were measured using a scanning electron microscope (SEM) (Thermo Fisher Scientific FIB-SEM GX4). The transmission electron microscopy (TEM) was examined to investigate the structural characterization using JEM-2100. The thermogravimetric analysis of the polymers were measured on a Netzsch STA449C TG/DSC thermal analyzer under nitrogen atmosphere between 20 $^{o}C$ and 800 $^{o}C$. Surface areas and pore size distributions were measured by Nitrogen isotherm adsorption-desorption at 77.3 K using ASAP 2460-3 (Micromeritics) volumetric adsorption analyzer. The UV-Vis absorption spectroscopy of polymers were measured via Shimadzu UV-2550 spectrophotometer. The fluorescence properties of samples were measured by fluorescence spectrometer (FLS1000) at the excitation wavelength of 544 nm. Time-resolved fluorescence decay spectroscopy was measured using a time correlated single-photon counting system (FLS1000).

**Electrochemical measurements**

Cyclic voltammetry (CV), electrochemical impedance spectra (EIS) and Mott-Schottky plot were measured on a CHI660E (Chenhua, Shanghai) electrochemical workstation with a standard three-electrode system (the sample modified Pt-disk electrode (Φ 3 mm) as the working electrode, Pt flake and Ag/AgCl as the counter electrode and reference electrode, respectively. The working electrode was prepared by the mixture slurry of photocatalyst (10 mg), isopropyl alcohol (1 mL) and

30 μL nafion. Mixture slurry (10 μL) was dropped on the platinum plate electrode and dried under an infrared lamp before the measurement. Cyclic voltammetry (CV) measurement was carried out with a scan rate of 100 mV $s^{-1}$ in argon atmosphere. A 0.1 M tetrabutylammonium hexafluorophosphate (MeCN-$NBu_4PF_6$) solution was used as the electrolyte in acetonitrile. EIS experiments were performed with a frequency range from 0.01 Hz to 100 k Hz, and $Na_2SO_4$ aqueous solution (0.5 M) was used as the electrolyte. The Mott-Schottky curve was measured at 1500, 2000, and 2500 Hz frequencies, the electrolyte was also 0.5 M $Na_2SO_4$ aqueous solution.

**Transient photocurrent measurements**

The transient photocurrent responses (*I-t*) was also measured on CHI660E (Chenhua, Shanghai) electrochemical workstation in a standard three-electrode system with Pt flake as the counter electrode and Ag/AgCl as the reference electrode. A 0.5 M $Na_2SO_4$ aqueous solution was used as the electrolyte. The polymer (10 mg) were added into 1 mL isopropyl alcohol and 30 μL Nafion mixed solution, and the working electrodes were prepared by dropping the mixture slurry (20 μL) onto an ITO glass (1 cm × 1 cm) electrode surface and dried under an infrared lamp before the measurement. Then the working electrode was irradiated under 300 W Xe lamp with cutof flter ($\lambda$ > 420 nm) irradiation with 20 s light on-off cycles.

**Photocatalytic hydrogen evolution**

The photocatalytic HER of BTPT-CMP1, BTPT-CMP2 and BTPT-CMP3 were conducted under visible light irradiation provided by a 300 W Xe lamp (CEL-HXF300) with a 420 nm cut-off filter. Typically, 50 mg of samples was suspended in mixture solution containing 50 mL $H_2O$ and 10 mL triethanolamine (TEOA), then 60 μL of chloroplatinic acid as co-catalyst was added into solution. After that, the system was degassed prior to photocatalysis in the ultrasound bath for 20 min. Then,

the temperature of reactant solution was sustained at 10 °C with flowing cool water during the reaction. The obtained gases were analyzed by gas chromatography every 0.5 h.

**AQY measurements**

Hydrogen evolution experiments for quantum efficiency was measured with monochromatic light obtained by using band pass filter of 405, 420, 450, 500, 550 and 630 nm with the intensities of 35.8, 37.2, 40.2, 49.4, 50.4 and 42.9 mW $cm^{-1}$, respectively. The AQY at a given wavelength was calculated as following equations.[1]

$$\mathrm{AQY} = \frac{2\mathrm{N0}}{\mathrm{Np}} \times 100\% = \frac{2 \times \mathrm{M} \times N_A \times \mathrm{h} \times \mathrm{c}}{\mathrm{S} \times \mathrm{P} \times \mathrm{t} \times \lambda} \times 100\%$$

Where M is the amount of $H_2$ (mol), $N_A$ is Avogadro constant ($6.02 \times 10^{23}$ $mol^{-1}$), h is the Planck constant ($6.626 \times 10^{-34}$ J/s), c is the speed of light in vacuum ($3 \times 10^8$ m/s), S is the irradiation area (19.6 $cm^2$ in our experiment), P is the intensity of irradiation light ($W/cm^2$ ), t is the irradiation time (1 h), λ is the wavelength of the monochromatic light (m).

**Synthesis of 2,4,6-Tris(5-bromothiophene-2-yl)-1,3,5-triazine (BTPT)[2]**

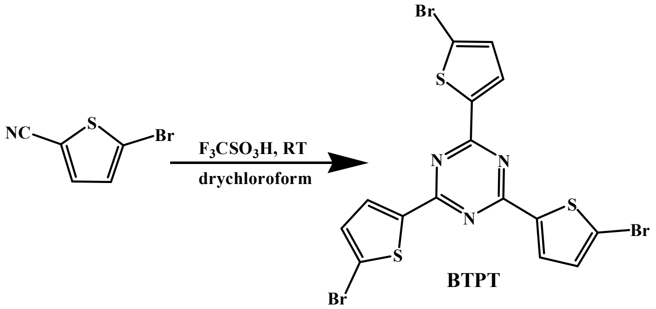


4.0 g (21.3 mmol) of 5-bromothiophene-2-carbonitrile was dissolved in 500 mL of dry chloroform, and then 12.8 g of (85.2 mmol) of trifluoromethanesulfonic acid was dropped into the solution at at 0 °C. The resultant solution was magnetically stirred for another 2 hours at 0 °C, and then, the temperature of the solution was risen to room temperature for 48 h. The mixture was rinsed with distilled water, and dried by anhydrous magnesium sulphate. The solution was obtained by

filtration. And then the solvent was distilled off by vacuum distillation. The solid as crude product was purified with recrystallization in toluene, affording the product as white needle. $^{1}$H NMR (500 MHz, Chloroform-*d*), δ, 7.97 (d, *J* = 4.0 Hz, 3H), 7.17 (d, *J* = 4.0 Hz, 3H). $^{13}$C NMR (126 MHz, $CDCl_3$), δ, 142.40, 136.54, 134.56, 130.10, 129.47, 127.46, 127.35, 126.82, 124.74.

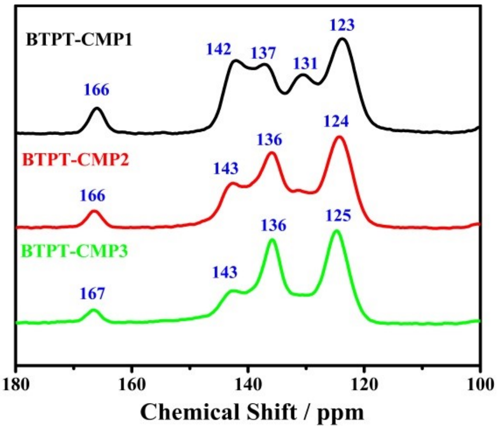


**Fig. S1**. Solid-state $^{13}$C CP-MAS NMR spectra of polymers.

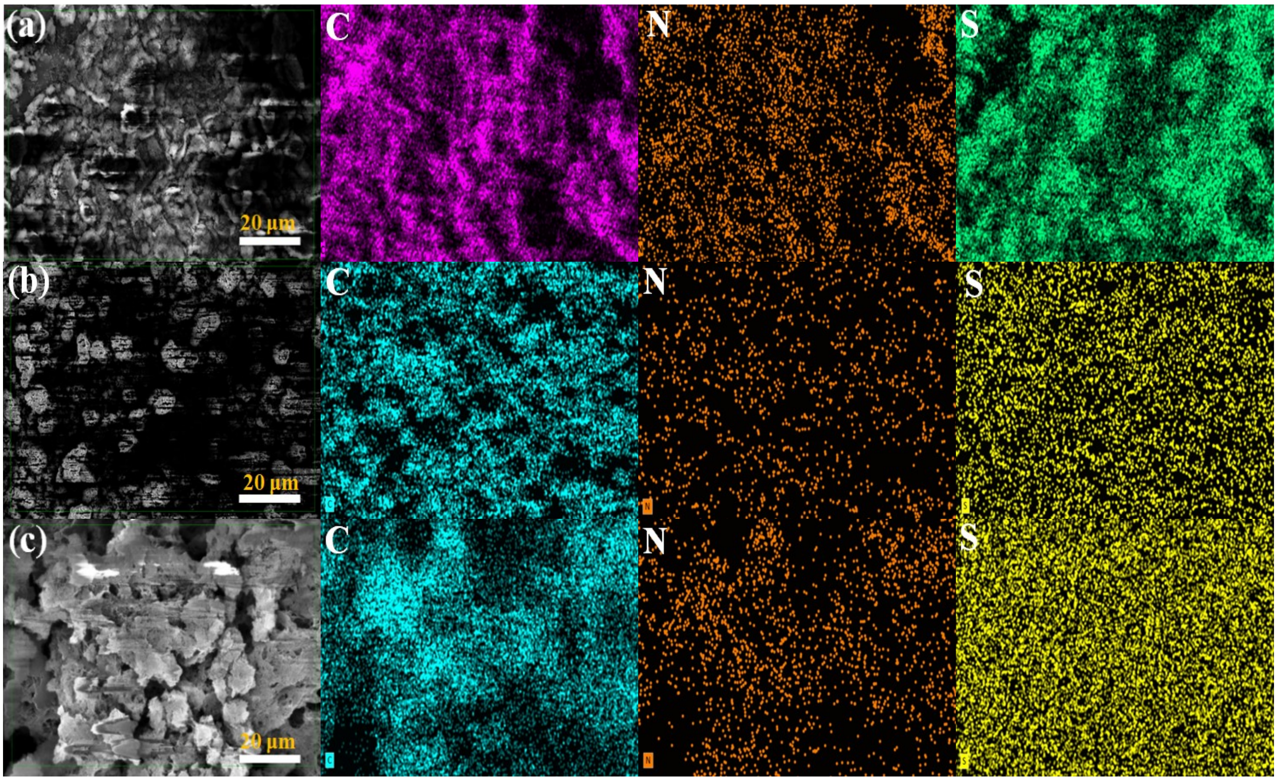


**Fig. S2.** Element mapping images for (a) BTPT-CMP1; (b) BTPT-CMP2; (c) BTPT-CMP3.

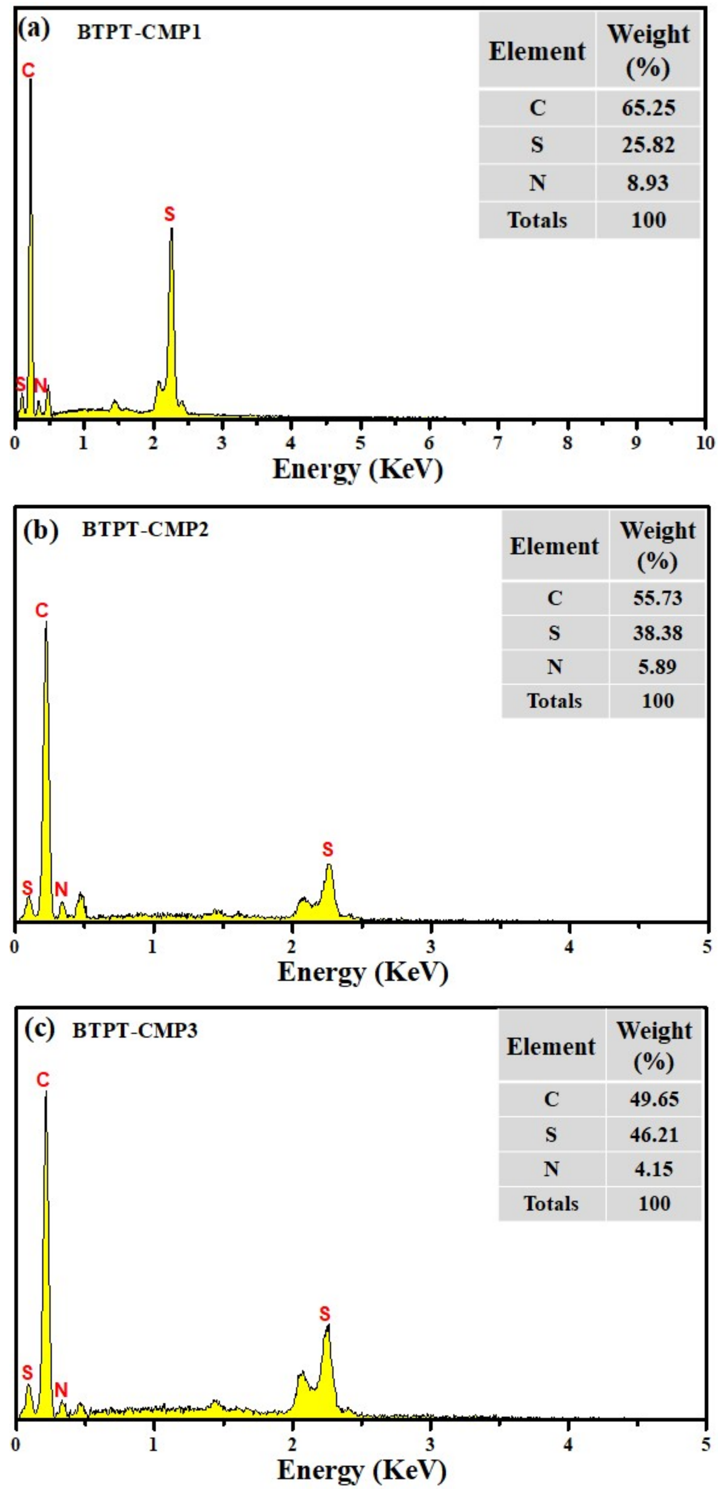


**Fig. S3.** EDS images of (a) BTPT-CMP1, (b) BTPT-CMP2 and (c) BTPT-CMP3.

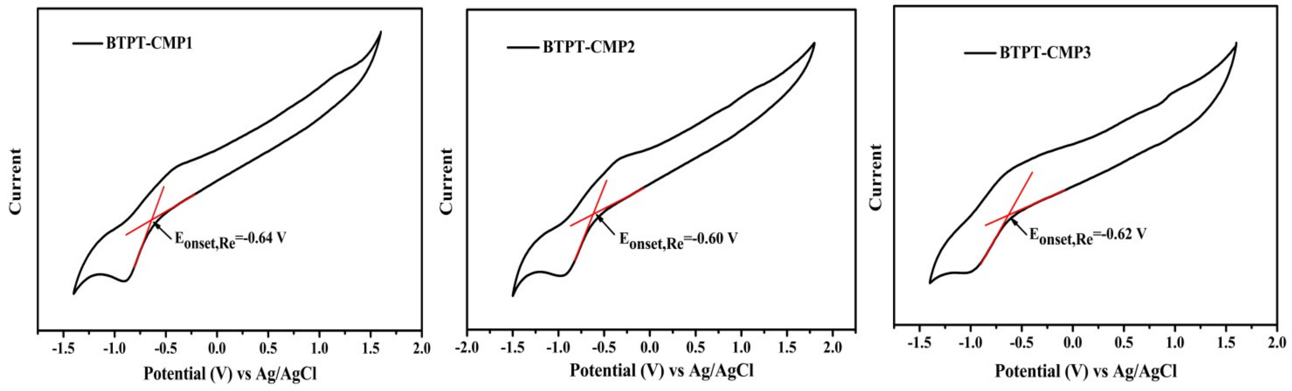


**Fig. S4.** CVs of the three polymers in MeCN-$NBu_4PF_6$ (0.1 M) at the scan rate of 100 mV $s^{-1}$ .

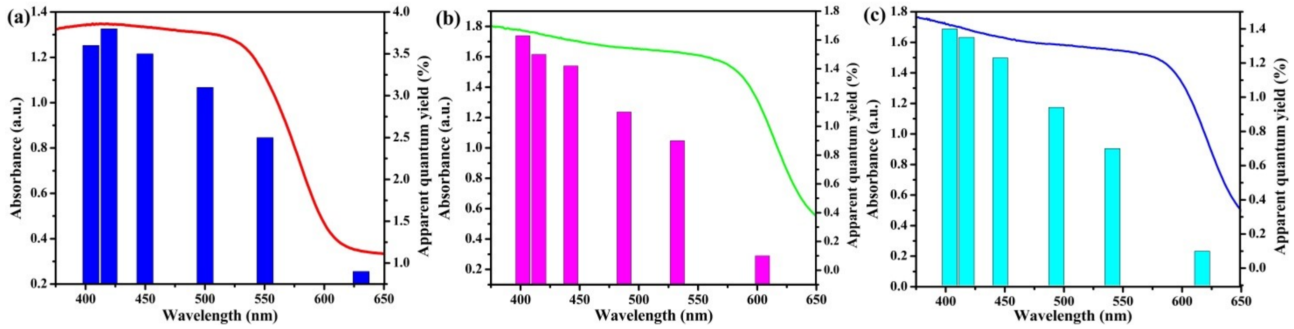


**Fig. S5.** Apparent quantum yield (AQY) of BTPT-CMP1 (a), BTPT-CMP2 (b) and BTPT-CMP3 (c) at various incident light wavelengths.

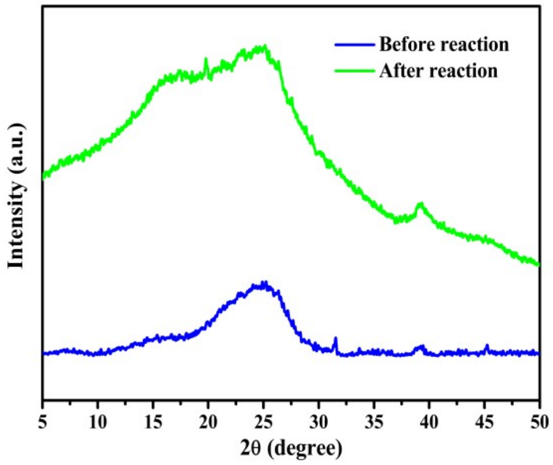


**Fig. S6.** XRD patterns of BTPT-CMP1 before and after irradiation under visible light ($\lambda$ > 420 nm).

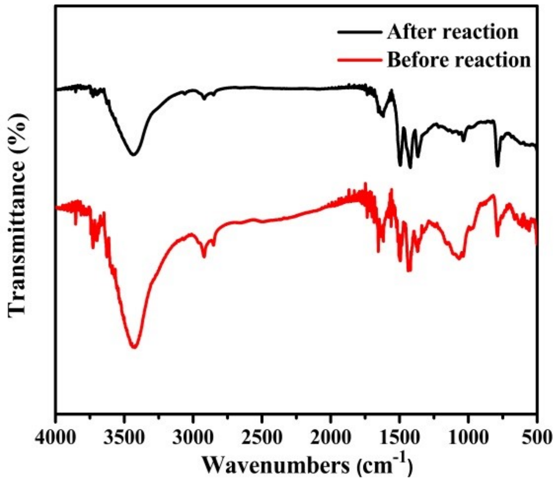


**Fig. S7.** FT-IR spectra of BTPT-CMP1 before and after irradiation under visible light ($\lambda > 420$ nm).

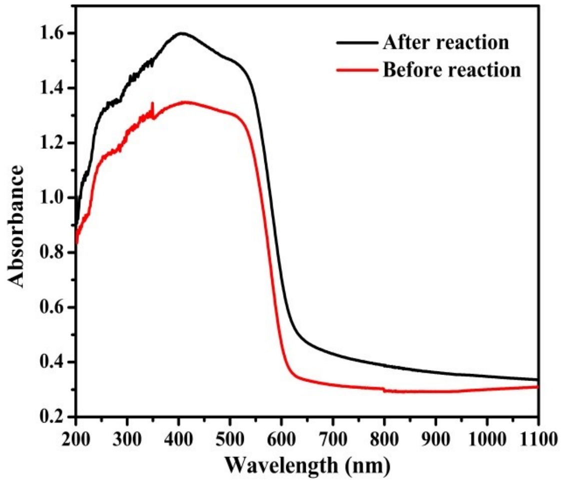


**Fig. S8.** UV-Vis diffuse reflectance spectra of BTPT-CMP1 before and after irradiation under visible light ($\lambda > 420$ nm).

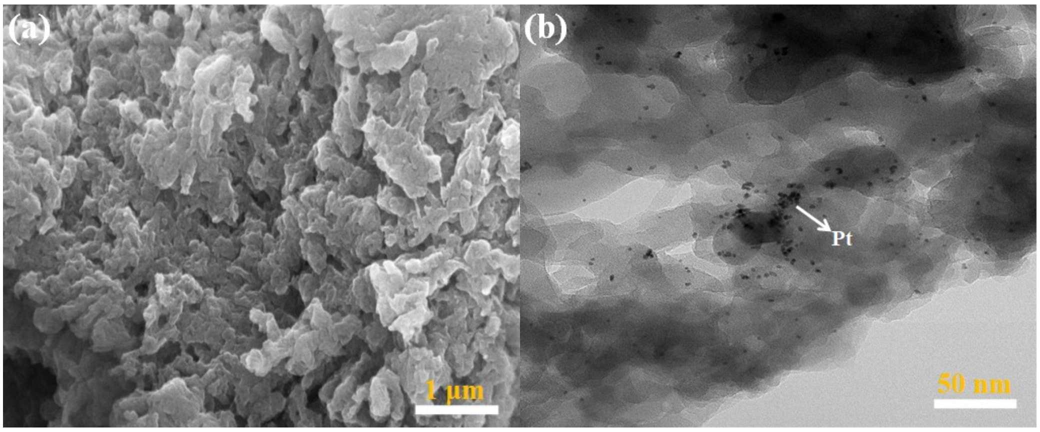


**Fig. S9.** SEM (a) and TEM (b) images of BTPT-CMP1 after irradiation under visible light ($\lambda > 420$ nm).

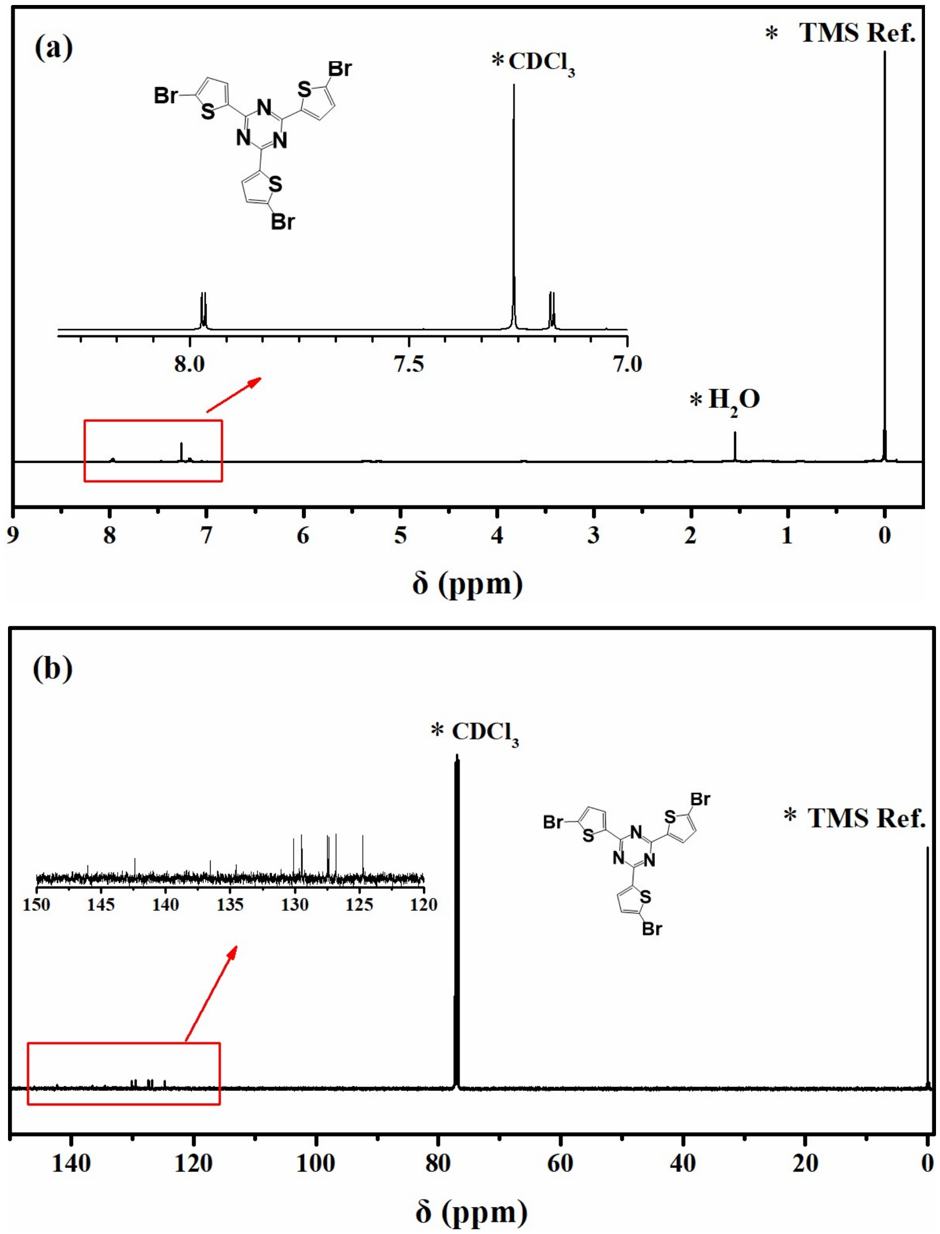


**Fig. S10**. (a) $^{1}H$ NMR spectrum of BTPT, (b) $^{13}C$ NMR spectrum of BTPT.

**References**

(1) W.Y. Huang, Z. Q. Shen, J. Z. Cheng, L. L. Liu, K. Yang, X. R. Chen, H. R. Wen and S. Y. Liu, *J. Mater. Chem. A.*, 2019, 7, 24222-24230.

(2) X. Xue, J. M. Luo, L. Q. Kong, J. S. Zhao, Y. Zhang, H. M. Du, S. Chen and Y. Xie, *RSC Adv.*, 2021, 11, 10688-10698.
